# Two Flat-Backed Polydesmidan Millipedes from the Miocene Chiapas-Amber Lagerstätte, Mexico

**DOI:** 10.1371/journal.pone.0105877

**Published:** 2014-08-27

**Authors:** Francisco Riquelme, Miguel Hernández-Patricio, Arnulfo Martínez-Dávalos, Mercedes Rodríguez-Villafuerte, Maira Montejo-Cruz, Jesús Alvarado-Ortega, José L. Ruvalcaba-Sil, Luis Zúñiga-Mijangos

**Affiliations:** 1 Posgrado en Ciencias Biológicas, Universidad Nacional Autónoma de México, Ciudad Universitaria, D.F., México; 2 Instituto de Física, Universidad Nacional Autónoma de México, Ciudad Universitaria, D.F., México; 3 Subcoordinación de Inventarios Bióticos, Comisión Nacional para el Conocimiento y Uso de la Biodiversidad, Tlalpan, D.F., México; 4 Facultad de Ciencias, Universidad Nacional Autónoma de México, Ciudad Universitaria, D.F., México; 5 Instituto de Geología, Universidad Nacional Autónoma de México, Ciudad Universitaria, D.F., México; 6 Museo del Ámbar Lilia Mijangos, San Cristóbal de las Casas, Chiapas, México; Sars International Centre for Marine Molecular Biology, Norway

## Abstract

Two species of fossil polydesmidan millipedes (Diplopoda: Polydesmida) embedded in amber are described from Miocene strata near Simojovel, in the Chiapas Highlands, Mexico. *Maatidesmus paachtun* **gen. et sp. nov**., placed into Chelodesmidae Cook, 1895, and *Anbarrhacus adamantis* **gen. et sp. nov**., assigned in the family Platyrhacidae Pocock, 1895. Morphological data from fossil specimens have been recovered using 3D X-ray micro-computed tomography and regular to infrared-reflected microscopy. Both fossil species are recognizable as new primarily but not exclusively, by collum margin modification and remarkable paranotal and metatergite dorsal sculpture.

## Introduction

The ecology, life history, physiology, morphology and phylogeny of millipedes (class Diplopoda) have been comprehensively reviewed in a few classical texts [1]–[3]. Millipedes have successfully adapted to soil and litter habitats in all subarctic climates. They contribute largely into soil cycles from both temperate and tropical forest. The fossil preservation of millipedes is very unusual mostly because they have terrestrial habits and non-recalcitrant tissues and cuticles. The oldest fossil record of millipedes is from the Mid-Silurian rocks in Scotland [4]. The geological record of Diplopoda is summarized elsewhere [1]–[3]. Accordingly, there is a large gap in the Mesozoic fossil record with the exception of the spirobolid millipedes from the Late Cretaceous of Mongolia and the presumably millipede *?Xylobius mexicanus* Müllerried, 1942 from the Late Jurassic/Mid-Cretaceous of Central Mexico [5], [6]. However, most members of the extinct taxa are notably found in Fossil Lagerstätten, as seen in the Late Carboniferous ironstone nodules from Britain and Cenozoic amber from Europe and Middle America [7]–[11].

The wedge-pushing type millipedes are generally represented by the order Polydesmida, whose fossil record dates back to the Paleogene of Europe in Baltic amber [8], [9]. Several polydesmidan millipedes from the younger deposits at the Neogene Middle America have also been recorded in Dominican Republic amber [10]. Millipedes from Miocene aged Chiapas amber (ca. 23-15 Ma.), Mexico, which has similar geological ages, sedimentary environments and paleobotanical affinities with Dominican amber, are only known for a recently-described stemmiulid species [11]. In this study, two fossil polydesmidan millipedes from the Chiapas amber are named and illustrated.

**Figure 1:**
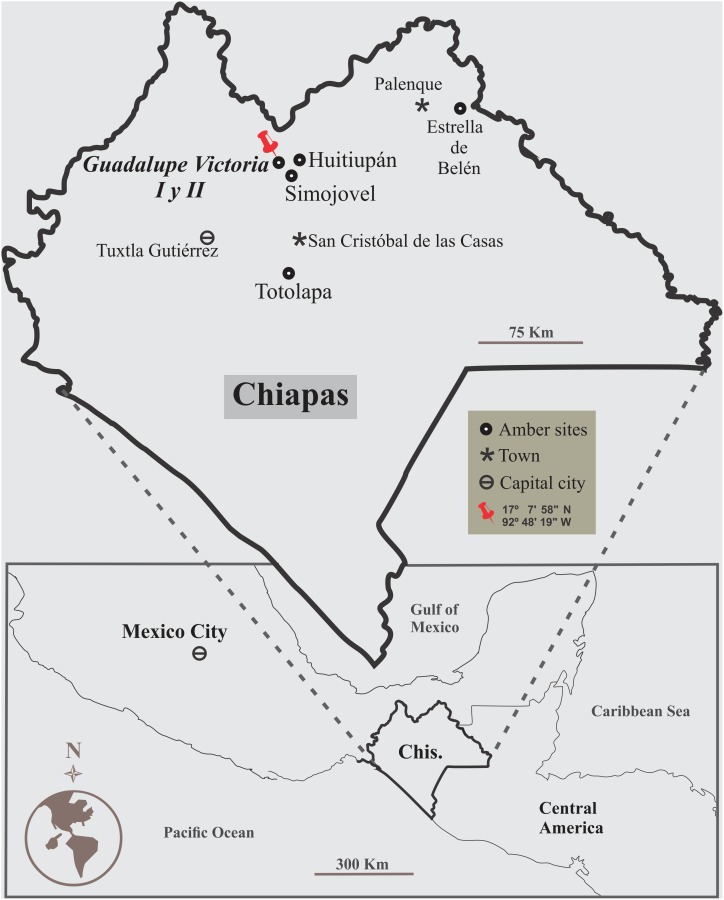
Location of the amber sites: Guadalupe Victoria I and II, Municipality of Simojovel de Allende, Chiapas, southern Mexico.

The fossil material studied has been now recovered from two private collections with geographic references. As a result of field geology and chemical provenance analysis, we track the source of millipede-embedded amber to the rocks of the Guadalupe Victoria site, near Simojovel in Chiapas, México (Fig. 1). Morphological data was collected by microimaging of fossil specimens using a nondestructive 3D X-ray micro-computed tomography (CT) and high-resolution microscopy with regular light to infrared-pass lens. The X-ray micro-CT scanning of a millipede specimen, as presented here, is also the first demonstration about the possibilities to recovery 3D images of fossil arthropods embedded in Chiapas amber using a laboratory-made technology, as alternative to the use of a synchrotron light source.

**Figure 2:**
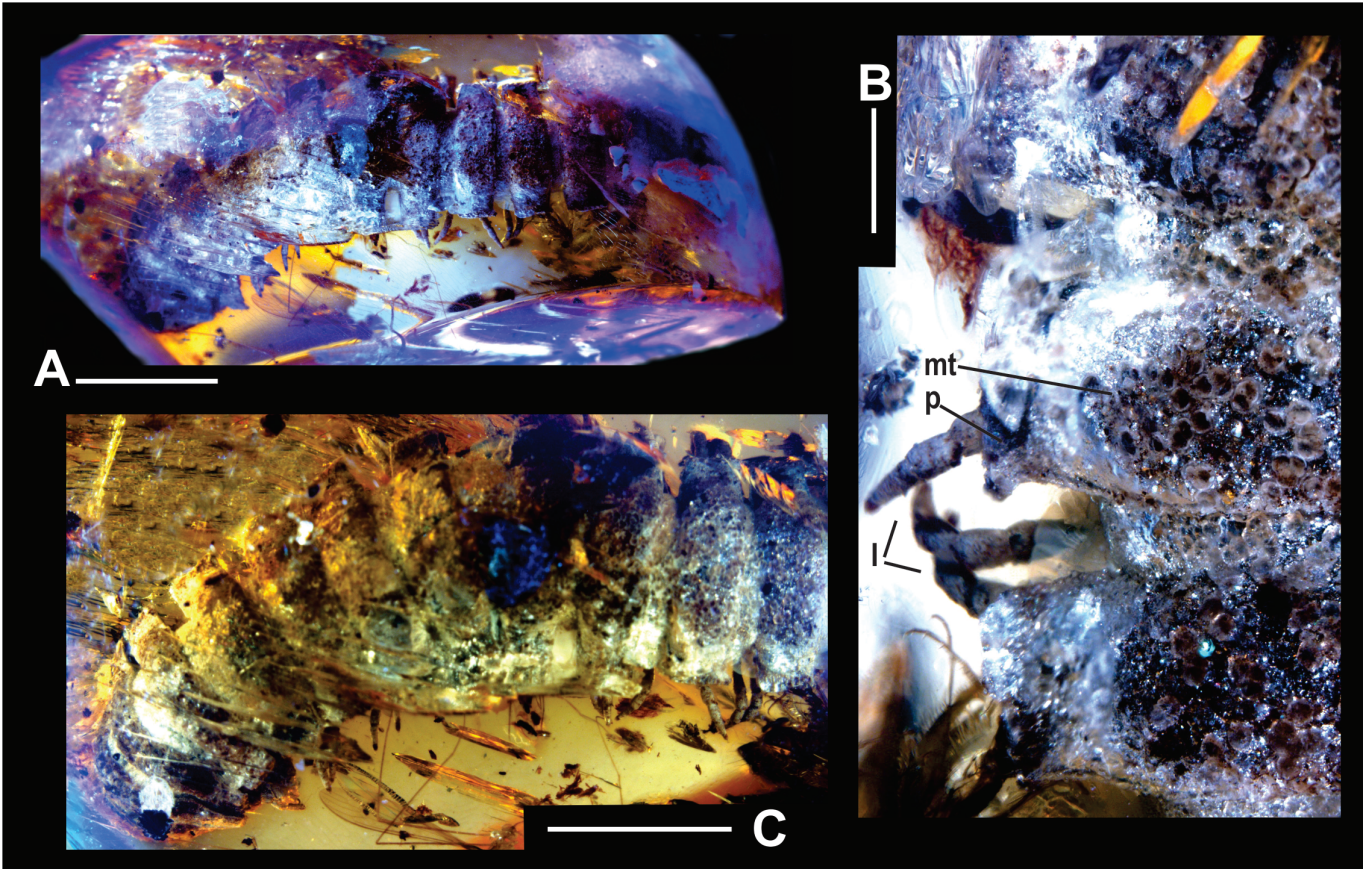
*Maatidesmus paachtun* nov. gen. and sp., holotype. (A) General view, scale bar 5 mm. (B) Close dorsal view of first segments, scale bar 1 mm. (C) Close dorsal view of metatergite and paranota in mid-body rings, scale bar 5 mm. See anatomical abbreviations in the main text.

**Figure 3:**
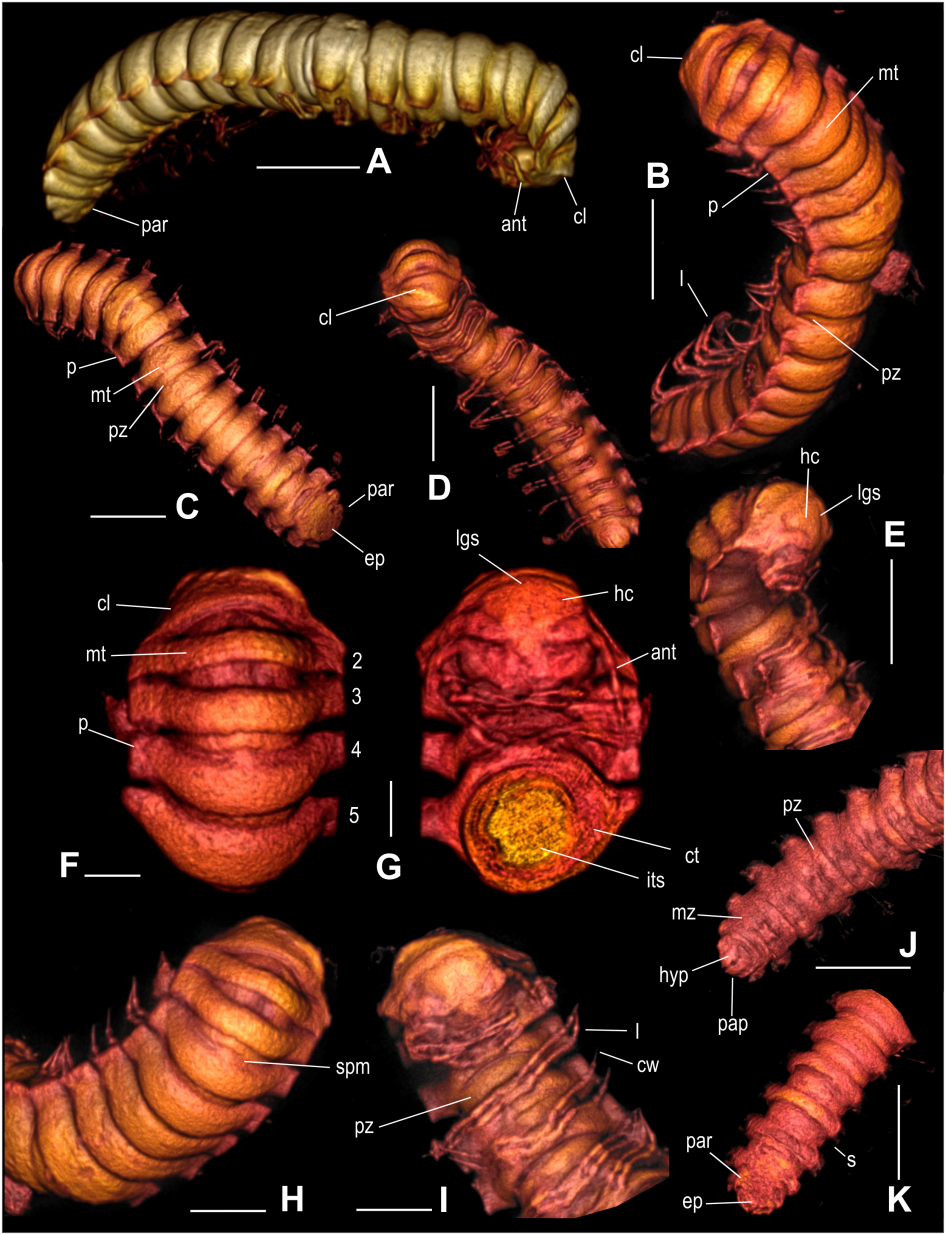
*Maatidesmus paachtun* nov. gen. and sp., holotype, 3D micro-CT reconstruction. (A–D): General view, scale bar 5 mm. E) Head, collum and first segments in lateral view, scale bar 5 mm. (F) Dorsal view of first segments, scale bar 2 mm. (G) Ventral view of head with a cross section of first segments, scale bar 2 mm. (H) Collum, metaterga and paranota in dorsal view, scale bar 3 mm. (I) Head and legs in dorsal view, scale bar 3 mm. (J) Last segments in ventral and dorsal view (K), respectively, scale bar 5 mm. All 3D images are expressed in virtual colors. See anatomical abbreviations in the main text.

**Figure 4:**
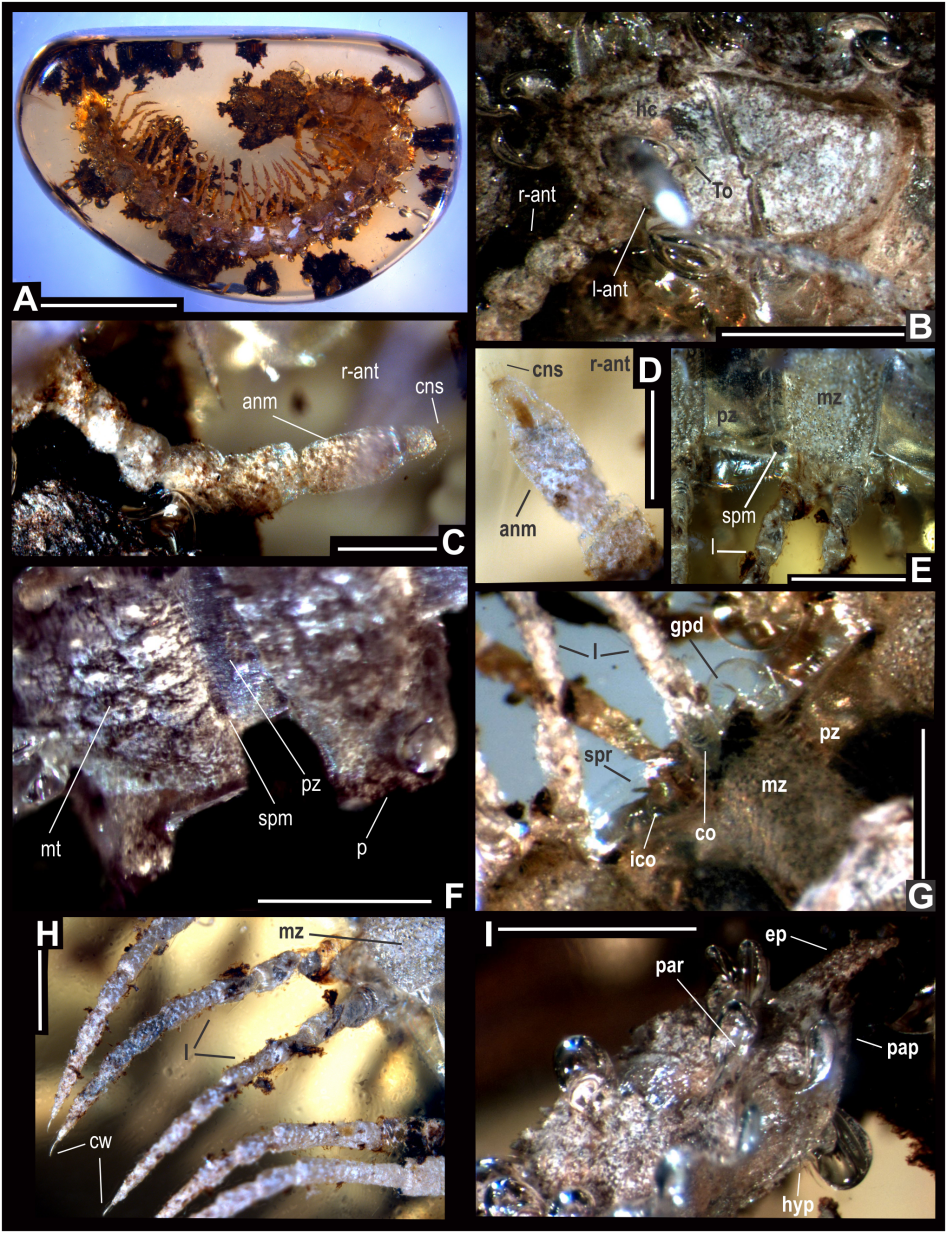
*Anbarrhacus adamantis* nov. gen. and sp., holotype. (A): General view, scale bar 5 mm. (B): Head, collum and antenna, scale bar 1 mm (C): Right antenna, scale bar 0.5 mm. (D): Antennomeres 6–8 and cones in right antenna, scale bar…mm. (E): Lateral view of rings 4–5, scale bar 0.5 mm. (F): Dorsal view of paranota and metatergite in 6–7 rings, scale bar 1 mm. (G): Lateral view of gonopods in 7 ring, scale bar 0.5 mm. (H): Legs, scale bar 0.5 mm. (I): Telson, scale bar 1 mm. See anatomical abbreviations in the main text.

**Figure 5:**
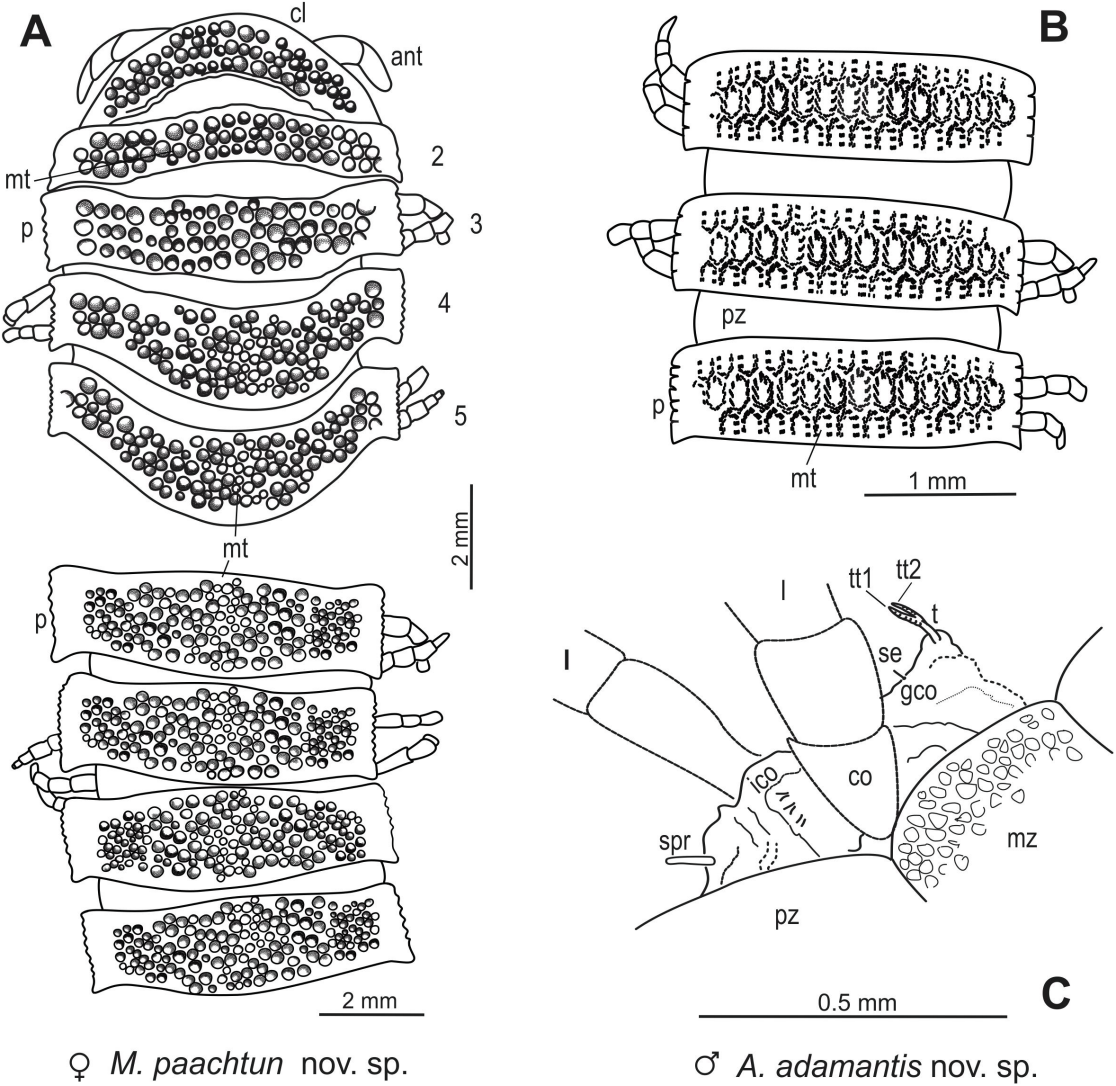
*Maatidesmus paachtun* nov. gen. and sp., schematic reconstruction, dorsal view. (A) collum, paranota and metatergite in rings 2–5 and mid-body rings. *Anbarrhacus adamantis* nov. gen. and sp., schematic reconstruction, dorsal view: (B) paranota and metatergite in rings 3–5. (C): inmature gonopods in ring 7. See anatomical abbreviations in the main text.

Two new, extinct genera *Maatidesmus* and *Anbarrhacus* and two new species are described herein. The fossil genus *Anbarrhacus* has been assigned to the family Platyrhacidae Pocock, 1895 and *Maatidesmus* to Chelodesmidae Cook, 1895. Currently both fossils species *Maatidesmus paachtun* **gen. et sp. nov**. and *Anbarrhacus adamantis* **gen. et sp. nov**. have been defined by available somatic characters (Fig. 2, 3, 4, 5). *A. adamantis* shows an exposed gonopods in situ on ring 7; however, it represents a stadium 7 male with relatively inmature gonopods, from which the gonopodal characters cannot be unambiguously assessed. To our knowledge, there are no previous formal descriptions of polydesmidan millipedes in the Chiapas amber, whose fossil record in the Neogene of Middle America provides some insight into origins and dispersal of New World Polydesmida millipedes from the Neotropics of North America to South America.

### Geological setting

The Guadalupe Victoria site near the town of Simojovel in Chiapas, Mexico, is characterized by carbonate and terrigenous sequences that resemble those exposed in the amber outcrops at the La Pimienta and Los Pocitos sites, also close to Simojovel [11]–[15]. The amber section consists of organic-rich lignite lenses, interbedded shales and coarse-fine grained sandstones with abundant iron oxides and pyrite nodules. The amber-bearing beds surrounding Simojovel primarily belong to the Mazantic shale and Balumtum sandstone strata dated as early to middle Miocene [11]–[15]. Preliminary, another outcrop with amber lumps was assigned to the Late Oligocene La Quinta unit [13]. However, the occurrence of this outcrop that presumably contains amber has not been recently verified in the field.

The amber-bearing rocks are the results of the nearshore and lowland sedimentation at the edge of the Chiapas Thrust–Fold Belt, which formed the Mountains of Chiapas spanning from the end of the Oligocene to mid-Pliocene [16]. Several amber localities sorted in the Mountains of Chiapas near Simojovel, Huitiupán, Totolapa, and Palenque (Estrella de Belén), they constitute an Amber Lagerstätte with extraordinary fossil preservation (Fig. 1). Taphonomy of arthropods, plants and microorganisms embedded in amber show a unique preservation of hard/soft tissues suggesting that organic decay was drastically interrupted [17]. Occurrence of small-sized vertebrates is infrequent and they are less preserved.

Palynology on amber sediments demonstrates that it represents a mangrove-like environment [18]. It is generally accepted that Chiapas amber was deposited in a subtropical forest with a Neotropical *Hymenaea* tree species recognized as the plant source [19]–[20]. Additionally, the biogeochemical data using synchrotron-based infrared microspectroscopy and organic mineral nomenclature of Chiapas amber has also been reviewed in a recently published contribution [21].

## Material and Methods

Fossil specimens studied here come from the amber pits known as the Guadalupe Victoria site, also known as “La Guadalupe” [11], which includes two indigenous communities (predominantly Tzeltal and Tzotzil): Guadalupe Victoria I and II, near the town of Simojovel, Chiapas, southern Mexico (Fig. 1). Both specimens are preserved in golden-yellow amber with glossiness from translucent to cloudy. Crude amber pieces with embedded millipedes were collected by anonymous indigenous miners. Specimen LZ.MALM.28 is currently housed in the public collection of the Museo del Ámbar Lilia Mijangos (MALM), San Cristobal de las Casas, Chiapas, Mexico. This collection is formally certified by the Instituto Nacional de Antropología e Historia (INAH) and curated by the present researchers (Riquelme, Montejo-Cruz and Hernandez-Patricio s. str.). Specimen IGM.4544 (Instituto Geológico Mexicano) is now housed in the Colección Nacional de Paleontología, Instituto de Geología, Universidad Nacional Autónoma de México (IGL-UNAM). No specific permits were required for the specimen description and paleontology fieldwork. A provenance analysis was carried out in amber samples using Fourier Transform Infrarred (FTIR) mico-spectroscopy; the IR spectra are available from authors upon request.

### 3D X-ray micro-computed tomography scanning

X-ray microtomography images were acquired using a benchtop micro-CT built at the Physics Institute (IF), UNAM. A detailed description of the equipment is presented elsewhere [22]. The micro-CT system is based on an Oxford Instruments Apogee XTG5011 tungsten anode X-ray tube with a nominal focal spot size of 35 µm, coupled to a Rad-icon Shad-o-Box 2048 flat panel detector (Teledyne DALSA Inc.). The projection image data were collected at 50 kVp, 1 mA with an integration time of 500 ms per frame and 360 degree orbit in 1 degree steps. The images were corrected for flat-field non-uniformities, dead-pixels, and dark noise. The Feldkamp-Davis-Kress algorithm [23], a Hamming filter with 0.7 cut-off frequency and an in-house developed program written in MATLAB Release 2010b (The MathWorks, Inc.) were applied for tomographic reconstruction. Finally, the open source programs ImageJ [24] and OsiriX [25] were used for 3D image post-processing and displaying.

### Photomicrographs and drawings

Regular and infrared-reflected photomicrographs were acquired using an apochromatic zoom micro-system combining regular and infrared-pass lens with LED and tungsten lamps and multiple image superimposition from >26 planes per image, as seen in [17]. Schematic drawings were hand traced by electronic pen using stereomicroscope, micrographs and CorelDraw X6 for graphic processing.

### Anatomical Abbreviations

anm, antennomere; ant, antennae; cl, collum; co, coxa; cns, cones, ct, cuticle; cw, claw; ep, epiproct; gco, gonocoxae; gpd, gonopods; hc, head capsule; hyp, hypoproct; its, inner tissues; ico, intercoxal process; l, leg; lgs, longitudinal sulcus of the head capsule; mt, metatergite; mz, metazonite; p, paranota; pap, paraproct; par, preanal ring; pz, prozonite; s, trunk segment; se, seta; spr, spiniform accessory projection; spm, suture between the prozonite and metazonite; st, stipes; t, telopodite; To, Tömösváry organ; tt, apical lobes of telopodite; anatomical elements of the right and left sides are denoted as (r) and (l), respectively (Fig. 2, 3, 4, 5).

### Terminology

The pattern of description and terminology follow [26]–[30]. Anatomical measurements are expressed in millimeters.

### Nomenclatural Acts

The electronic edition of this article conforms to the requirements of the amended International Code of Zoological Nomenclature, and hence the new names contained herein are available under that Code from the electronic edition of this article. This published work and the nomenclatural acts it contains have been registered in ZooBank, the online registration system for the ICZN. The ZooBank LSIDs (Life Science Identifiers) can be resolved and the associated information viewed through any standard web browser by appending the LSID to the prefix “http://zoobank.org/”. The LSID for this publication is: urn:lsid:zoobank.org: pub: 0096A7D5-0826-4BCE-907A-80476A24F77C. The electronic edition of this work was published in a journal with an ISSN, and has been archived and is available from the following digital repositories: PubMed Central, LOCKSS.

## Results and Discussion

### Systematic Paleontology

Class **Diplopoda** Blainville, in Gervais, 1844.

Subclass **Chilognata** Latreille, 1802/1803.

Infraclass **Helminthomorpha** Pocock, 1887.

Order **Polydesmida** Pocock, 1887.

Suborder **Leptodesmidea** Brölemann, 1916.

Family **Chelodesmidae** Cook, 1895.

***Maatidesmus*** Riquelme et Hernández **gen. nov.**

ZooBank LSID: urn:lsid:zoobank.org:act:BB03262D-6BA7-48B4-8FA8 A06CFE7E0A01.

#### Etymology:

Derived from the Maya word *maat*- (means “amber”) and -*idesmus,* which is a common suffix in the Chelodesmidae.

#### Diagnosis:

As for the only known species below.

#### Type species:

***Maatidesmus paachtun*** Riquelme et Hernández sp. nov.

Designated by monotypic species. Fig. 2–3, 5A. Movie S1.

ZooBank LSID: urn:lsid:zoobank.org:act:AFA5FAA8-12E3-4D0E-A09D-62078F640DF3.

#### Etymology:

The specific epithet *paachtun* means *“stony-backed”,* composed from the Maya elements*: paach-: “back”* plus *tun*: “stone”, refers to conspicuous, lobulated dorsal sculpture in collum and metatergite in rings 2–5.

#### Holotype:

LZ.MALM28, and only known specimen. An entire adult female, three-dimensionally preserved (Fig. 2–3.).

#### Horizon and locality:

The amber-bearing beds at the Guadalupe Victoria site, Latitude 17° 07′ 58′ ´N, Longitude 92° 48′ 19′ ´ W (Fig. 1), near the town of Simojovel, State of Chiapas, México. These rocks belong to Mazantic shale and Balumtum sandstone strata dated as early-middle Miocene, ca. 23-15 Ma [11]–[15].

#### Diagnosis:

Large-sized chelodesmid, adult female, with head+19 rings, 35.5 mm total length. Head wider than collum, vertex moderately granulated, with a deep vertigial sulcus extending from the top of antennal sockets; antenna clavate, long and robust, antennomere length relationships: (2, 6) > (3) > (4, 5) > (1, 7). Collum with a convex dorsum covered with margin lobations, metatergite in rings 2–5 heavily lobulated in three transverse rows arrangement; whereas metatergite in rings 6 and 7 with an asymmetric pattern of swellings rather than truly-lobed. Differs from extant representatives of the Chelodesmidae by its conspicuous, coarsely lobulated dorsal sculpture in collum and metatergite in rings 2–5. Paranota on all rings short, inflated, subrectangular, dorsally roughened, tilted toward the body midline, with tick, minute, acute margins. In the longitudinal direction, dorsally, paranota+metatergite in rings 2–3, 17, 18 and 19 distinctly narrower than width of the rest of the rings. Epiproct wide, short, triangular, with caudal edged broadly blunt. Monotypic.

#### Description:

*– General characteristics*: Fossil specimen LZ.MALM.28 represents an adult female with the entire trunk and head preserved (Fig. 2A–B). Distinguished by its conspicuous, coarse, lobulated dorsal sculpture in collum and metatergite in rings 2–5 (Fig. 2C, 3B–H, and 5A). Head wider than collum (Fig. 3G–I). Trunk composed of 19 segments with paranota and telson, ca. 35.5 mm in length, 7.9 mm in maximum width, and W/L ratio of 22.2% (Fig. 3A–D). Rings 2–5 equal to collum in overall width, whereas posterior ring widths slightly increasing gradually to ring 17, thence gently narrowing over the last rings and telson (Fig. 3C–D, 3K, and 3J); Paranota inflated, subrectangular, dorsally roughened, tilted toward the body midline (Fig. 2B–C and 3A–G). Epiproct wide, short, triangular (Fig. 3C). Since amber is cloudy around female genitalia and this is a tiny structure, it cannot be clearly assessed.

#### Taphonomic features:

a partial dorsal portion of rings 18–19 were accidentally scraped during amber polishing by collectors; accordingly, the preanal ring was scarcely altered in dorsal view, affecting the epyproct surface dorsally. The head is ventrally bent as seen in a defensive position. The body decay was dramatically interrupted by rapid polymerization of plant resin, which preserved intact soft tissues inside the fossil as shown in a cross section of inner body layers from the reconstructed X-ray tomography (Fig. 3G); although the cuticle is slightly recrystallized due to reacting with amber, it also preserves colored morphology. Copious embedded biodebris along and around the body, mostly insect parts from mosquitos and ants; there are also plant fragments and soil particles. The piece of amber is golden to orange yellow with translucent to cloudy glossiness, internally recrystallized with abundant transverse, small fractures (Fig. 2).

#### Coloration preserved in amber:

Ground color in head and rings creamy white to nut-brown splatter (Fig. 2). All rings show slightly dissolved and recrystallized portions of cuticle as crystal-white patches, probably calcium carbonate salts. Head and antenna white. Collum whitish colored, most of the lobes nut-brown. Paranota generally from crystalline to creamy white, metaterga also nearly white with nut-brown lobes; upper part of prozona white and lower side creamy gray to nut-brown (Fig. 2C). Sterna pale to nut-brown. Legs white, ending in a nut-brown splashes (Fig. 2C).

#### Head:

convex, slightly wider than collum, with a vertex moderately granulated, and deep vertigial sulcus extending from the top of antennal sockets; sockets slightly impressed ventrolaterally; frons elevated above level of clypeus and labrum. Cardo, stipes and gnathal lobe are prominent. Antenna clavate, long and robust, thicker than legs; antennomeres length relationships: (2, 6) > (3) > (4, 5) > (1, 7), apical cones are hardly distinguished (Fig. 3G–E, and 3I).

#### Collum:

strongly convex anteriorly in dorsal view, with anterior margins broadly rounded, not hiding the head in lateral view; dorsal surface conspicuously lobed posteriorly, with posterior margins angled (Fig. 3D–F, and 3H).

#### Trunk:

composed of 19 rings with paranota and telson (Fig. 3A–D). In the transverse direction, rings 2–5 equal to collum in overall width, consecutive ring widths increase gradually to ring 16, thence gently narrow over the last rings (17–19) and telson (Fig. 3C, 3D, 3K, and 3J); In the longitudinal direction, dorsal view, paranota+metatergite in rings 2 and 3 distinctly narrower than width of paranota+metatergite in consecutive rings, which gradually increase reaching a maximum width in mid-region and moderately decrease to ring 16, thence in rings 17–19 are as narrow as 2 and 3; between metazonite and prozonite waist striated; the prozonite surface almost smooth, metazonite surface finely granulated. Paranota on all rings short, inflated, subrectangular, dorsally roughened, tilted toward the body midline, with thick, minute, acute margins, and distinctly but irregularly toothed (Fig. 2C, 3B–C, 5A). The mid-dorsal surface of metatergite with at most three transversal rows of lobes in rings 2–5, whereas rings 6 and 7 with swellings randomly located (Fig. 2C, 5A); pleural tubercles conspicuous; sternites surface moderately setose; ozopores opening laterally as seen in rings 5, 7, 9–10, 12–13, and 15–16; there are not differences between segments with pores and without pores. Legs extended ventrally in all rings, long, slender, with elongated femora and tarsi, ending in a short claw. The podomere length relationships: femur > tarsus >prefemur > (postfemur, tibia) (Fig. 2C, 3B, and 3I).

#### Telson:

Epyproct wide, short, triangular, with caudal edge broadly blunt, not extended beyond paraprocts due to taphonomic alteration; paraprocts semicircular, surface finely granular but the edges are smoother and scaly; hypoproct triangular but sharply truncate, posterior margin convex, apex acute, with setae on each side of the midline (Fig. 3C and 3J–K).

#### Remarks:

– After the Paradoxosomatidae, the family Chelodesmidae is one of the most diverse polydesmidan group comprising 230 extant genera with nearly 450 species [31], whose distribution now extends to Africa, Middle America and South America [28]. At the present time, there are five living species of the Chelodesmidae recorded in Mexico assigned to the genera *Chondrodesmus, Rhaphandra* and *Eutyporhachis*, the latter is also present in the Chiapas Highlands [32]–[33]. *Maatidesmus paachtun* **gen. et sp. nov**. has in common several characters with the living representatives of the Chelodesmidae, including the shape of collum, paranota and telson, but differs by the conspicuous, coarsely lobulated dorsal sculpture in collum and metatergite in rings 2–5 (Fig. 2C, 3B–H, 5A). *M. paachtun* is the first fossil species of this family from the Neogene of Middle America. Other fossil chelodesmid form has been previously known from Dominican amber based on headless specimen [10].

### Systematic Paleontology

Superfamily **Platyrhacoidea** Pocock 1895.

Family **Platyrhacidae** Pocock, 1895.

***Anbarrhacus*** Riquelme et Hernández **gen. nov.**

ZooBank LSID: urn:lsid:zoobank.org:act:B1FD30A1-B55D-4205-8E99-C17F34386661.

#### Etymology:

Derived from the Arabic voice *ánbar* (means “amber”) and –*rhacus,* which is a common suffix in the Platyrhacidae.

#### Diagnosis:

As for the only known species below.

#### Type species:

***Anbarrhacus adamantis*** Riquelme et Hernández **sp. nov**.

Designated by monotypic species. Fig. 4.

ZooBank LSID: urn:lsid:zoobank.org:act:DF667E57-51C8-4104-BC95-B2375DF3E5E4.

#### Etymology:

The specific epithet *adamantis* is derived from *adamantus* (Latin): “diamond”, refers to dorsal sculpture in collum and metatergite with a rhomboidal-pattern (Fig. 4 and 5B–C).

#### Holotype:

IGM.4544, and only known specimen. Stadium 7 male, three-dimensionally preserved, almost complete; only left antenna is partially missing (Fig. 4).

#### Horizon and locality:

Amber-bearing beds at the Guadalupe Victoria site, Latitude 17° 07′ 58′ ´N, Longitude 92° 48′ 19′ ´ W, near Simojovel de Allende, State of Chiapas, Mexico (Fig. 1). These rocks belong to Mazantic shale and Balumtum sandstone strata dated as early-middle Miocene [11]–[15].

#### Diagnosis:

Small-sized Platyrhacidae, stadium 7 male **w**ith head+17 rings, 19.8 mm total length. Head moderately convex, rough, and setose, wider than collum. Antenna clavate, long, copiously covered with setae, antennomeres length relationships: 5>6> (2≈3≈4) > (1≈7), with four long, slender sensory cones. Distinguished by its granulated dorsal sculpture in paranota and metaterguite; paranota on all rings, wide, granulated, with thick, rounded margin, waist laterally striated; metatergite coarsely granulate, mid-dorsally ornamented in a rhomboidal-pattern, all minute tubercles and setae multiple. Intercoxal process between legs in ring 7, irregular with distal, spiniform accessory projection. Gonopods immature, small, in situ on ring 7, gonocoxae bulbous, slightly crooked, with a long distal setae on medial surface; gonopodal telopodite shrunken, bipartite, apical lobes of the tibiotarsus laminate, striated and acute. Monotypic.

#### Description:

*– General characteristics*. Fossil specimen IGM.4544 represents a stadium 7 male, which preserves the entire trunk and head with apical portion of left antenna missing (Fig. 4). Head convex, wider than collum (Fig. 4B). Trunk composed on 17 segments with paranota and telson, ca. 19.8 mm in length, 2.8 in maximum width, W/L ratio of 14.1%. Rings increasing gradually in width from collum to posterior 2/3 of the body and gently narrows over the last rings and telson; epiproct fairly long and spathulate (Fig. 4I). Paranota wide and granulate, metatergites ornamented in a rhomboidal-pattern (diamond-like) in mid-dorsal region (Fig. 4F). Legs long and slender, extended lateroventrally (Fig. 4H). Although relative immature, gonopods are exposed, small, in situ on trunk ring VII, gonocoxae bulbous, crooked, gonopodal telopodite shrunken, bipartite, apical lobes of the tibiotarsus minute, laminate, striated and acute (Fig. 4C and 5C).

#### Taphonomic features:

left antenna partially broken, antennomeres 3–6 accidentally missing by polishing amber (Fig. 4B). Empty molds of bubbles are present all over the dorsal portion of the head and trunk; particularly, in the margins of paranota (Fig. 4A–B and 4I). It seems that these bubbles were produced as result of dissolved water vapor from soils during rapid amber hardening (polymerization). Decaying of carcass was drastically interrupted by resin polymerization; because of this the hard/soft tissues and true color morphology have been preserved (Fig. 4). Head and trunk slightly bent ventrolaterally; body randomly surrounded by blackish to brown soils and plant debris. The piece of amber is golden to citrine yellow with translucent glossiness, in a pebble-like shape and roughly polished (Fig. 4A).

#### Coloration preserved in amber:

Most of the head and antenna from cream colored to nearly white, 6th antennomere of right antenna colorless, setiferous tubercles blackish-brown, clypeus darker, a conspicuous black patch above the Tömösváry organ, labrum pale yellowish (Fig. 4B–D). Collum gray to white, blackish in midline (Fig. 4B). Paranota generally cream colored with metaterga whitish gray; upper surface of prozona blackish gray and lower sides brownish. Sterna pale yellowish to brown and legs creamy white to pale yellowish (Fig. 4E–H).

#### Head:

moderately convex, slightly wider than collum, densely covered with minute setae; surface in the vertex, frons and vertigial sulcus roughened; clypeus sparsely setose. Gnathochilarium with short median expansion, labral surface with several flat papillae, cardo, stipes, and gnathal lobe are appreciably well-defined. The Tömösváry organ is conspicuous below the antenna and between the incisura lateralis (Fig. 4B). Antenna clavate, long, copiously covered with setae, antennal length 2.5 mm. The antennomeres length relationships: 5>6> (2≈3≈4) > (1≈7), with four long, slender sensory cones; 5^th^ and 6^th^ antennomeres with long sensitive setae near apex (Fig. 4B–D).

#### Collum:

broadly rounded anteriorly, almost semicircular, laterally articulated with the rear of the head capsule, not hiding the head; dorsal surface granulated, with small rounded tubercles and setose, posterior margins irregular and rounded (Fig. 4B).

#### Trunk:

Composed of 17 rings with paranota and telson (Fig. 4A). Trunk rings generally similar on structure, prozonite and metazonite separated by a well-defined striated waist (Fig. 4E–F). Metatergite mid-dorsally surface rough, granulated, with polygonal furrows in a rhomboid pattern, all setiferous tubercles multiple (Fig. 4F and 5B). Rings 16 and 17 with posterior margin of metatergite coarsely granulate. Pleural tubercles on all rings are conspicuous, variable in size. Prozonite surface polished, lightly smooth (4E–F). Paranota on all rings wide, arising low on body and slightly declined, with posterior margin rounded. Paranota dorsal surface roughened, in middle paranotal process with copious granules on anterior angle nearly rectangular; paranota lateral margins distinctly but irregularly toothed; anterior margin lightly convex, posterior lightly concave (Fig. 4F); paranota in rings 11 and 12 with posterior angle acute and not lateral marginal thickening. The posterior edge of the paranota and metatergite dorsally striated (Fig. 4F). Ozopore open dorsally in rings11 and 12, hard to distinguish in other rings. Sternites surface moderately setose, as wide as long. Spiracles small and pyriform as seen on ring 6. Legs extended lateroventrally on all rings, slender with elongated femora and tarsi, ending in a long claw; relative lengths of podomere are as a follows: femur >tarsus >prefemur > (postfemur, tibia) (Fig. 4H). Intercoxal process between legs in ring 7, irregular with distal, spiniform accessory projection (Fig. 4G).

#### Telson:

Preanal ring with several large setae, epiproct spathulate in outline, fairly long, extending beyond the paraprocts, with caudal edged rounded-triangular and slightly acute; paraproct semicircular, each with setae; hypoproct sharply truncated (Fig. 4I).

#### Gonopods:

relatively immature, small, in situ on ring 7; gonocoxae bulbous, slightly crooked, with a conspicuous setae on medial surface, posterior margin in plate greater almost the height of the sternites, gonopod telopodite thin, short, shrunken, bipartite, apical lobes of the tibiotarsus minute, laminate, striated and acute; gonopore inconspicuous due to body position (Fig. 4G and 5C).

#### Remarks:

*– Anbarrhacus adamantis* **gen. et sp. nov**. is a stadium 7 male, consequently, the putative apomorphic characters from the male gonopods cannot be assessed unequivocally. The living species are typically diagnosed using male/female genitalia [26]–[27], [30], [34]–[35]. Other living species of the Platyrhacidae described upon female specimens have been considered species inquirenda [34]. At the present, there is no satisfactory treatment of platyrhacidan species that includes fossils in the available literature. Probabilities of finding adult male specimens with preserved, exposed genitalia in the fossil record are extremely low. In this context, *A. adamantis* shares somatic characters with several extant species of the family Platyrhacidae as seen in the genera *Nyssodesmus, Psammodesmus, Exallostethus, Platyrhacus and Hoffmanorhacus* [27], [34]–[36]. Taxonomical affinities include the shape of collum, paranota, metatergites, epyproct, others, as described above. Thus, the specific relationships at the genus and species level of *A. adamantis* with living platyrhacidan millipedes are indicated by somatic characters; but distinguished from them by four long, slender sensory cones in antenna and the conspicuous, granulated dorsal sculpture in paranota and metaterguite. Also differs from them by the intercoxal process among legs in ring 7.

The extant representatives of Platyrhacidae comprise a greater diversity of about 37 genera and 180 species. Those are distributed in the Indo-Australian region from Myanmar to the Solomon Islands [37]; whereas the New World members of Platyrhacidae are found in the Neotropics from the southernmost Mexico to northern Brazil [30], [33]–[36]. Here nine genera were proposed by Cook, 1895 [38], platyrhacids from South America have been reviewed by Chamberlin, 1941 [39] and platyrhacids from southern México, West Indies and Central America have been studied in significant works [34]–[36].

There are few living species of the Platyrhacidae recorded in México, i.e. *Exallostethus trinax* Hoffman, 1975 collected in the Chiapas Highlands [26]. Other species such as the first-described living forms of *Platyrhacus,* i.e. *P. bilineatus* and *P. mexicanus* Lucas, 1840 have been considered species inquirenda [34]. Thus, *A. adamantis* represents the first fossil species of the Platyrhacidae in the southernmost part of North America.

## Conclusion

The 3D X-ray micro-CT scanning has revealed many of the diagnostic body parts of chelodesmid millipede embedded in cloudy, fractured amber with copious biodebris along and around the body. The micro-CT imaging also shows intact soft tissues inside the fossil chelodesmid, which is the first evidence of ancient soft tissue preservation in millipedes at any geological time. This demonstrates the potential of 3D X-ray microimaging using a lab-made technology. To our knowledge, such technique is first applied in the Chiapas amber arthropods.

On the other hand, several polydesmidan forms have been detected in the Chiapas amber samples belonging to private collections; they also contain many unidentified specimens (Riquelme, Hernandez-Patricio and Montejo-Cruz pers. obs.). Enthusiastic amber trading has produced a large gap in the fossil record of the Chiapas amber paleobiota, including Diplopoda. Accordingly, all fossil millipede species embedded in amber collected from Miocene strata in the Mountains of Chiapas are currently new to science. Both *M. paachtun* and *A. adamantis* share close affinities with their extant congeners and respective families. Millipedes show a primitive and very conservative morphology, younger fossil and living forms resemble those in the Late Paleozoic. In general terms, the fossil millipedes in the Chiapas amber are typically modern forms, but future findings and descriptions of new polydesmidan millipedes will probably show highly variable fossil forms at genus and species level only.

The Polydesmida fossil record is notoriously incomplete for global diversity analysis. However, the occurrence of *M. paachtun* and *A. adamantis,* which have been placed in extinct genera and restricted to Neogene of the Middle America amber, provides some additional insights into Polydesmida phylogeny and current Neotropical distribution from the southernmost North America to northern South America.

## Supporting Information

Movie S1 *Maatidesmus paachtun* **gen. et sp. nov**. 3D X-ray micro-computed tomography.
(AVI)
